# Assessment of Pregnant Women’s Knowledge and Perceptions of Antenatal Ultrasound in Saudi Arabia

**DOI:** 10.3390/healthcare12141409

**Published:** 2024-07-15

**Authors:** Sami A. Alghamdi, Najwa A. Dhahi, Fahad A. Gashash, Ghasan F. Abuturboush, Afaf A. Hazzazi, Ali B. Alhailiy, Yazeed Alashban

**Affiliations:** 1 Radiological Sciences Department, College of Applied Medical Sciences, King Saud University, Riyadh 145111, Saudi Arabia; aldhahinajwa@gmail.com (N.A.D.); yalashban@ksu.edu.sa (Y.A.); 2 Department of Radiology and Medical Imaging, King Saud University Medical City, Riyadh 145111, Saudi Arabia; fahadgashash@gmail.com (F.A.G.); gabutarboush@ksu.edu.sa (G.F.A.); 3 Radiology Department, Maternity and Children Hospital, Dammam 32253, Saudi Arabia; afaf.hazzazi@yahoo.com; 4 Department of Radiology and Medical Imaging, College of Applied Medical Sciences, Prince Sattam bin Abdulaziz University, Al Kharj 11942, Saudi Arabia; a.alhailiy@psau.edu.sa

**Keywords:** knowledge, perception, antenatal ultrasound, pregnant women, Saudi Arabia

## Abstract

**Background:** This study aims to evaluate pregnant women’s knowledge of antenatal ultrasound in Saudi Arabia and its correlation with demographic factors like age and education to enhance prenatal care. **Methods:** A cross-sectional study was conducted in six Saudi Arabian hospitals, involving 22 questions split between sociodemographic information and knowledge of antenatal ultrasound. Descriptive statistics were used to characterize the participants’ demographics and responses. Additionally, inferential statistics were employed to analyze the relationships and differences among the study variables. **Results:** Among the 531 pregnant women in the study, most demonstrated a good understanding of antenatal ultrasound, identifying its various uses. Specifically, they recognized its roles in evaluating fetal growth (82.5%), placental location (81.7%), amniotic fluid volume (67%), and fetal morphology (65%), predicting the delivery date (79%), and determining the baby’s sex (89%). A majority viewed ultrasound as important (89.3%), safe (82.3%), and tolerable (76.3%) for prenatal care. Additionally, 66.7% felt adequately informed, mainly through clinical staff and doctors. Younger age, lower education, lack of prior ultrasound experience, and first pregnancy were linked to lower knowledge. Approximately 65% were uncertain about the nonionizing radiation properties of ultrasound. **Conclusions:** The study found that while most pregnant women in Saudi Arabia understand the objectives of antenatal ultrasonography, there are gaps in their knowledge about its nonionizing properties. Younger age, lower education, lack of prior ultrasound experience, and first pregnancy contribute to lower knowledge.

## 1. Introduction

Prenatal care plays a pivotal role in ensuring the health and well-being of expectant mothers and developing fetuses. Antenatal ultrasound, a noninvasive method and a crucial component of contemporary prenatal care, provides valuable insights into fetal development, aiding in the early detection of potential complications [1]. Clinical data have shown that it also enhances the efficacy of clinical administration [2]. Moreover, obstetric ultrasonography plays a crucial role in identifying the existence and position of a pregnancy, confirming the viability of the embryo, estimating the gestational age and due date, evaluating the fetal presentation, determining the location of the placenta, assessing the amniotic fluid, and examining the fetal anatomy [3].

Multiple studies have demonstrated that pregnant women possess a substantial understanding of the utilization of ultrasound during pregnancy [4,5,6,7]. This can be attributed to the widespread implementation of ultrasound as a standard aspect of antenatal care as well as the unrestricted availability of information [5]. Studies have demonstrated that pregnant women are also knowledgeable about the specific applications of ultrasonography [4,7,8]. The tasks included in these studies were aimed at evaluating pregnancy duration, approximating the expected delivery date, identifying any fetal abnormalities, tracking fetal growth, determining fetal sex, and confirming the occurrence of pregnancy or multiple pregnancies. Pregnant women’s primary sources of information on obstetric ultrasound, as identified by previous research, are health workers [4,6,8], family, friends, and social media [9].

Pregnant women tend to perceive prenatal ultrasonography as a highly secure medical practice [2,7,10]. Nevertheless, some women have expressed reservations over the safety of ultrasound, proposing that an excessive number of ultrasound scans may have detrimental effects on newborns, potentially leading to cancer and substantial impairments in both mothers and their babies [5,6,9]. Several authors have advocated for providing health education to the general public regarding the safety of ultrasonography [2,5]. A study revealed that the majority of pregnant women are unaware of the safety of diagnostic ultrasound throughout the prenatal period [11]. 

The use of antenatal ultrasound in prenatal care is well established worldwide; however, its understanding and adoption can have a significant impact on maternal and fetal outcomes. Despite the existing body of research, there remain significant gaps in understanding the depth of knowledge and perceptions among pregnant women, particularly in specific cultural contexts such as Saudi Arabia. Previous studies in Saudi Arabia have been limited, with one study conducted in a single hospital, and did not consider the entire country’s regions or include both private and public healthcare sectors.

This study aims to meticulously assess the depth of understanding and perceptions among pregnant women in Saudi Arabia regarding antenatal ultrasound, examining how these perceptions correlate with various demographic factors. Understanding these factors is crucial for developing more effective prenatal care models and educational programs tailored to the specific needs and concerns of this population. 

## 2. Methodology

### 2.1. Participants and Recruitment

In this cross-sectional study, surveys were distributed across six diverse hospitals in Saudi Arabia from November 2023 to January 2024, including two private hospitals, one university hospital, and three maternity hospitals managed by the Saudi Health Ministry. The six hospitals included in the study were selected from five different provinces: Riyadh, Makkah, the Western Region, Asser, and Albaha. This selection was based on their geographic and demographic diversity to accurately reflect the experiences of the pregnant population across varied healthcare settings in the country. The aim was to gain a comprehensive understanding of the healthcare experiences of pregnant women throughout Saudi Arabia. 

A stratified sampling technique ensured variety in the patient sample, with stratification criteria based on hospital type (private, university, government), geographic provinces across Saudi Arabia, and demographic factors such as age and education level. Participants were categorized based on their employment status into the following categories: student, unemployed, housewife, freelance, employed, and retired. In this study, housewives were considered as a separate category from the unemployed, with the unemployed defined as those actively searching for jobs. 

To facilitate data collection, five undergraduate radiology students and six radiological technologists were trained as proficient interviewers for pregnant outpatients to enhance their understanding of respondents’ knowledge. After their ultrasound sessions, pregnant women at these hospitals were provided a quick response (QR) code to access the survey on their electronic devices, which they could complete online or on paper as per their preference. For those choosing the paper option, responses were manually entered into a secure database by trained data entry staff. Participation in the survey was voluntary, with participants informed about the study’s objectives and assured of their privacy protection.

### 2.2. Survey 

The questionnaire was first developed based on previous studies [12,13] and then adapted to fit the goals of our study. It was in Arabic and comprised 22 questions divided into two sections: (A) sociodemographic information, and (B) pregnant women’s knowledge of antenatal ultrasound

Ethical considerations were rigorously followed: The study was conducted in compliance with ethical standards and received approval from the institutional review board at King Saud University, Medical City (Ref. No. 23/0848/IRB). Informed consent was obtained from all participants before their involvement in the study.

### 2.3. Categorization of Knowledge Level of Ultrasound

To determine the two levels of ultrasound knowledge (adequate and inadequate), the 3-point responses (don’t know, yes, and no) were converted into scores by assigning a score of ‘1’ to the correct answer and ‘0’ to the other two responses for each of the 11 items. The total score of correct responses was 11; scores were sorted into two categories: ≤6 being an inadequate knowledge level and ≥7 being an adequate knowledge level. 

### 2.4. Statistical Analysis

The data were carefully analyzed using SPSS statistical software for Windows, version 26.0 (IBM Corp., Armonk, NY, USA). Descriptive statistics were employed to characterize the participants’ demographic features and responses. The Pearson chi-square test and odds ratios were used to assess and measure the association between the knowledge level of ultrasound (adequate or inadequate) and the categorical sociodemographic characteristics of the study subjects (age group, institution, education level, employment status, trimester stage, ultrasound before this pregnancy and first pregnancy. Multivariate binary logistic regression was used to identify the independent variables associated with the knowledge level of ultrasound. A *p*-value of ≤0.05 and 95% confidence intervals were used to report the statistical significance and precision of the results

## 3. Results

A total of 531 pregnant women participated in the survey. Table 1 illustrates the demographic distribution of the study participants. The age categories were segmented as follows: <20 years (11 participants), 20–29 years (132 participants), 30–39 years (217 participants), and 40+ years (171 participants). The distribution of the educational levels of the participants was varied: 59% of the participants had attained bachelor’s degrees, 22.6% had completed primary and secondary education, 7% held master’s degrees, 6% had diploma certificates, 3% had PhD qualifications, and a minimal proportion of 2.4% lacked any formal education. The majority of participants were employed (229 participants), and housewives constituted the next largest group (191 participants). Regarding pregnancy stages, women who did not know their trimester stage constituted the largest group (192 participants), and women in their third trimester formed the second largest group (155 participants). The distribution of pregnant women receiving care at private and public healthcare facilities was nearly even, at 48% and 52%, respectively. The majority of participants had undergone an ultrasound examination before their current pregnancy (410 participants), whereas 121 participants were experiencing their first pregnancy.

Table 2 presents an overview of pregnant women’s knowledge and understanding of ultrasound capabilities, specifically based on their responses to inquiries such as “Can ultrasound evaluate fetal growth?”. A total of 438 participants, accounting for 82.5% of the sample, recognized that ultrasounds can help assess fetal growth. Additionally, 434 (81.7%), 282 (53%), and 228 respondents (42.9%) were aware that ultrasounds can be used to evaluate placental location, identify chromosomal or genetic abnormalities, and diagnose structural abnormalities, respectively. Further, 79.80% and 89.4% of the participants knew that ultrasounds could help predict the delivery date and determine the baby’s sex, respectively. Moreover, when asked about the significance of ultrasound examinations in prenatal healthcare, 474 (89.2%) of the respondents expressed positive views. And concerning the risk of ultrasound, the question “Does diagnostic ultrasound use ionizing radiation?” was answered with “I do not know” and “Yes” by 357 (67.2%) and 48 (9%) participants, respectively, and the answer “No” was given by just 126 (23.7%) participants. Moreover, 437 respondents (82.3%) expressed their belief in the safety of ultrasounds for both mothers and fetuses. 

Table 3 presents a summary of the participants’ responses regarding their self-evaluation and sources of acquiring information about ultrasound. Over 65% of the participants reported that they possessed adequate knowledge (enough, good, or excellent) about antenatal ultrasound scans. From the multiple responses, we noticed that 76.3% of the participants responded “comfortable” to the question: “What do you think about ultrasound examinations?”. Only 3.4% responded ”painful”. Furthermore, by permitting multiple responses, 58.6% of the participants indicated that they received information about ultrasound from referring doctors, followed by radiology clinical staff (37.3%), the internet and social media (31.1%), family and friends (28.1%), and others (16.2%), with doctors and clinical staff being the primary sources of this information.

### 3.1. Association between Ultrasound Knowledge Level and Patients’ Sociodemographic Characteristics

Out of a total of 531 participants, 397 (74.8%) had an adequate knowledge level of ultrasound. Table 4 presents the association between participants’ knowledge levels and their sociodemographic characteristics. Notably, the participants’ age group, educational level, experience of having an ultrasound before this pregnancy, and first pregnancy are all statistically significantly associated with their knowledge level of ultrasound. For the age groups, the odds of participants aged 20–29, 30–39, and 40+ years having an adequate level of ultrasound knowledge are 14.06 times, 16.28 times, and 9.70 times higher, respectively, compared with participants aged less than 20 years (*p* = 0.005). For educational levels, the odds that participants with education levels of school, diploma, BSc, and MSc or PhD have an adequate level of ultrasound knowledge are 9.17, 23.83, 19.01, and 24.20 times higher, respectively, compared to participants with no education (*p* = 0.012). Additionally, participants who had an ultrasound before pregnancy are 3.25 times more likely to have adequate ultrasound knowledge compared to those who did not have an ultrasound before pregnancy (*p* < 0.00001). Moreover, participants reporting that this was not their first pregnancy have a 1.76 times higher likelihood of possessing adequate ultrasound knowledge compared to those for whom this was their first pregnancy (*p* = 0.004). The other characteristics of participants (institution, employment, and trimester stage) are not statistically significantly associated with their knowledge level of ultrasound (Table 4).

### 3.2. Independent Variables Associated with Ultrasound Knowledge Level

In the multivariable analysis, binary logistic regression using the forward Wald method was employed to identify the independent variables associated with an adequate knowledge level of ultrasound, based on the significant variables identified in the bivariate analysis. A model incorporating variables such as age group, educational level, ultrasound utilization prior to this pregnancy (yes), and first pregnancy status (no) was compared against a model with only a constant. The comparison was statistically significant, indicating that these variables as a set effectively distinguish between participants with inadequate and adequate knowledge levels of ultrasound (χ^2^ = 69.410; *p* < 0.001; DF = 9). The Hosmer–Lemeshow test, which assesses the goodness of fit for logistic regression models and serves as an alternative to the model chi-square test, yielded a value of 7.495 (*p* = 0.484; DF = 8). Given that the *p*-value exceeds 0.05, it is inferred that the model provides an acceptable fit to the data. This non-significance indicates that the model prediction does not significantly differ from the observed. Nagelkerke’s R^2^ of 0.18 indicates a moderate relationship between prediction and grouping. The Wald criterion demonstrated that the variables in the model at Step 4 (as presented in Table 4) made a significant contribution to predicting an adequate knowledge level of ultrasound. The final model validation was carried out using a classification table which summarizes the observed group and predicted group classification. The overall prediction success was 75.7% (19% for inadequate knowledge level and 96.4% for adequate knowledge level) (Table 5).

### 3.3. Association between Self-Evaluation and Knowledge Level of Ultrasound

The participants were asked to self-evaluate their knowledge of ultrasound on a 5-point scale. The responses were as follows: 30.1% rated their knowledge as excellent, 27.3% as good, 9.2% as enough, 23.5% as fair, and 9.8% as not enough. The distribution of these subjective responses was highly statistically significantly associated with the knowledge score levels adequate knowledge level and inadequate knowledge level of ultrasound. That is, a higher proportion of subjects who had responded “excellent”, “good”, “enough”, or “fair” for their self-evaluation knowledge were found to have an adequate knowledge level of ultrasound when compared with the participants who had responded ”not enough” for their self-evaluation knowledge (χ^2^ = 106.034, *p* < 0.0001) (Table 6).

These findings suggest that factors such as age group (20 to 29 years), literacy level, experience of having undergone ultrasound, and whether it is their first pregnancy influence the adequacy of their knowledge levels regarding ultrasound. Also, the participants’ self-evaluation is highly statistically significantly associated with their knowledge level, as obtained from their responses of different items related to ultrasound.

## 4. Discussion

Fetal ultrasound is widely recognized as a safe diagnostic tool, and studies conducted at diagnostic level have reported no adverse effects [12,14]. In the present study, the associations between pregnant women’s knowledge of ultrasound and various sociodemographic characteristics were explored. The findings of this study highlight the influence of sociodemographic factors on ultrasound among participants. Younger age, lower educational attainment, lack of prior ultrasound experience, and first pregnancy were associated with lower ultrasound knowledge levels. 

The significant observations from this study revealed a positive association between age and ultrasound knowledge; higher ages were markedly linked to enhanced antenatal ultrasound knowledge. Education level also had a considerable effect on ultrasound knowledge. Participants with higher levels of education, including diploma, BSc, and MSc and PhD, demonstrated substantially higher odds of possessing adequate ultrasound knowledge compared to those with no formal education. This result is consistent with the studies conducted by Abduljabbar et al. (2020) and Molla et al. (2021), which underscored the influence of educational attainment on ultrasound proficiency [15,16]. Mengistie et al. (2023) discovered that occupational roles, such as housewife, government employee, and private sector employee, were positively correlated with knowledge among Ethiopian women, and they also identified a beneficial link between primary education and positive attitudes [13]. These findings are in contrast to those of the present study as employment status did not exhibit a significant association with ultrasound knowledge in the study population. This discrepancy suggests that regional differences in sociodemographic characteristics and cultural contexts may play a role in shaping health-related knowledge levels. 

The study findings showed that a high percentage of pregnant women were aware of the various aspects of ultrasound. The majority of participants knew that ultrasound could be used to evaluate fetal growth, fetal morphology, and placental site, and to diagnose structural abnormalities. They also knew that ultrasounds could determine delivery time and fetal sex. However, there was a lack of knowledge regarding whether nonionizing radiation is used in diagnostic ultrasounds. These findings are consistent with those reported by Maniragena et al., who found that a notable number of subjects underscored ultrasound’s critical role in antenatal care, particularly for evaluating fetal health, estimating gestational age, determining fetal gender, estimating the expected delivery date, and aiding in delivery planning. However, awareness was limited concerning the use of ultrasound in detecting fetal abnormalities [17].

In the current study, the type of healthcare facility (public versus private) and gravidity status (primiparous versus multiparous) were factors that had no statistically significant impact on the attainment of knowledge related to ultrasound in antenatal care. These results are in contrast to those presented by Abduljabbar et al., which demonstrated a significant association between gravidity and knowledge score [15]. 

In the present research, over 80% of the participants comprehended the primary diagnostic uses of ultrasound, which is encouraging. This aligns with Dasan et al.’s results, which revealed that over 70% of pregnant women in India possess a comprehensive understanding of ultrasound’s role in assessing gestational age, determining the expected date of delivery, and detecting fetal anomalies [4]. Conversely, Molla et al. discovered that merely 35.3% of expectant mothers in Ethiopia possess adequate knowledge of obstetrical ultrasound scanning [16].

The present study’s findings showed that approximately 65% of the participants were uncertain about the nonionizing radiation properties of ultrasound. Nonetheless, a significant proportion of participants perceived ultrasound as important (89.3%), safe (82.3%), and tolerable (76.3%) for prenatal care. In studies conducted by Molla et al. and Saleh et al., 82% and 97% of female participants, respectively, considered ultrasound to be a safe practice for pregnant women [2,16].

In our study, over 65% of the participants indicated that they had adequate knowledge (ranging from enough to excellent) about antenatal ultrasound scans. Notably, the primary sources of this information were doctors and clinical staff. This finding underscores the significant role that healthcare professionals play in disseminating knowledge about antenatal care and the trust placed in them by pregnant women.

We recommend the adoption of longitudinal approaches in subsequent research to thoroughly investigate the progression of ultrasound awareness among pregnant women over time. In addition, including a more diverse and extensive group of participants would enhance the generalizability of the results. We also recommend exploring the effectiveness of different educational interventions in improving pregnant women’s understanding of ultrasound examinations and assessing the role of digital media and online resources in shaping expectant mothers’ perceptions and attitudes toward prenatal care, considering the increasing reliance on these channels for health information.

The primary limitation of this study was its cross-sectional design, which limited the ability to establish causality between sociodemographic factors and knowledge levels. The study’s timeframe, spanning November 2023 to January 2024, coincides with the winter season, characterized by lower temperatures. This period may influence prenatal care initiation patterns among pregnant women, particularly those in their first trimester. Concerns about the safety of ultrasound in early pregnancy, compounded by the cold weather, may deter some women from seeking early prenatal care. This seasonal factor likely contributes to the notably low percentage of participants in their first trimester. Hence, seasonal variations represent a potential bias that could affect the study’s outcomes. Further, the sample for the current study was drawn from only six hospitals located in various provinces of Saudi Arabia, potentially limiting its representativeness and the generalizability of the findings to diverse populations. The study did not differentiate between national and non-national participants, which could influence the results. 

To enhance the comprehensiveness of future research, it is suggested that the sampling framework is expanded to include a more extensive and diverse selection of hospitals across different geographic settings. Incorporating a mixed-methods approach that combines quantitative surveys with qualitative interviews can also provide deeper insights and corroborate the quantitative data.

## 5. Conclusions

Although this study confirmed that a substantial proportion of pregnant women in Saudi Arabia have a thorough comprehension of the essential objectives of antenatal ultrasonography in prenatal care, there were still gaps in their knowledge, specifically regarding their uncertainty about the nonionizing properties of ultrasound. Furthermore, it reveals how sociodemographic factors influence pregnant women’s ultrasound knowledge. Younger age, lower educational attainment, a lack of prior ultrasound experience, and first pregnancies are all associated with lower levels of knowledge. Nevertheless, participants perceived ultrasound as important, safe, and tolerable for prenatal care, indicating the significant role of healthcare professionals as primary sources of information. We recommend future longitudinal studies that encompass diverse participant groups and explore different educational interventions to enhance ultrasound understanding and improve prenatal care practices, with a focus on assessing the role of digital media and online resources in shaping pregnant women’s perceptions and attitudes toward antenatal care.

To enhance practice, healthcare professionals should receive continuous training to provide up-to-date, evidence-based information about antenatal ultrasound to pregnant women, emphasizing its safety and benefits. Implementing standardized educational sessions during prenatal visits can help address gaps in knowledge. In terms of policy, national guidelines should be developed to ensure consistent messaging about the safety and importance of antenatal ultrasound across all healthcare facilities. Encouraging collaboration between public and private healthcare sectors can create comprehensive educational programs accessible to all pregnant women. Regarding education, integrating antenatal ultrasound education into the curriculum for healthcare professionals is essential, ensuring they are well equipped to educate patients effectively. Utilizing digital media and online resources to disseminate accurate information about antenatal ultrasound can enhance understanding and address misconceptions.

## Figures and Tables

**Table 1:** Distribution of sociodemographic and pregnancy-related characteristics of study subjects (*n* = 531).

Characteristics	No. (%)
Age group (in years)	
Less than 20	11 (2)
20–29	132 (25)
30–39	217 (41)
40+	171 (32)
Institution	
Private	257 (48)
Public	274 (52)
Education	
None	13 (2.4)
Primary, Intermediate, and Secondary	120 (22.6)
Diploma	32 (6)
BSc	312 (59)
MSc	38 (7)
PhD	16 (3)
Employment	
Student	24 (4.5)
Unemployed	51 (10)
Housewife	191 (36)
Freelance	17 (3)
Employed	229 (43)
Retired	19 (3.5)
Trimester stage	
Don’t know	192 (36)
First	62 (12)
Second	122 (23)
Third	155 (29)
Ultrasound before this pregnancy	
Yes	410 (77)
No	121 (23)
First pregnancy	
Yes	126 (24)
No	405 (76)

**Table 2:** Distribution of pregnant women’s responses with respect to their knowledge of antenatal ultrasound.

Knowledge Items	Responses		
	Don’t Know	Yes	No
Can ultrasound evaluate fetal growth?	80 (15.1%)	438 (82.5%) (C) *	13 (2.4%)
Can ultrasound evaluate fetal morphology?	105 (19.8%)	350 (65.9%) (C) *	76 (14.3%)
Can ultrasound evaluate amniotic fluid volume?	121 (22.8%)	389 (73.3%) (C) *	21 (3.9%)
Can ultrasound evaluate the placental site?	86 (16.2%)	434 (81.7%) (C) *	11 (2.1%)
Can ultrasound diagnose chromosomal or genetic abnormalities (e.g., Down syndrome)?	163 (30.7%)	282 (53.1%) (C) *	86 (16.2%)
Can ultrasound diagnose structural abnormalities?	201 (37.8%)	228 (42.9%) (C) *	102 (19.3%)
Can ultrasound estimate the expected date of delivery?	77 (14.5%)	424 (79.8%) (C) *	30 (5.7%)
Can ultrasound predict the sex of the baby?	49 (9.2%)	475 (89.4%) (C) *	7 (1.4%)
Does diagnostic ultrasound use ionizing radiation?	357 (67.2%)	48 (9.0%)	126 (23.8%) (C) *
Is ultrasound safe for moms and fetuses?	82 (15.4%)	437 (82.3%) (C) *	12 (2.3%)
Do you think that an ultrasound examination is important in healthcare during pregnancy?	0 (0%)	474 (89.3%) (C) *	57 (10.7%)

* (C) correct answer.

**Table 3:** Distribution of patients’ self-evaluation and sources of information about ultrasound.

Items	No. (%)
How do you evaluate your knowledge about ultrasound and pregnancy?	
Not enough	52 (9.8)
Fair	125 (23.5)
Enough	49 (9.2)
Good	145 (27.3)
Excellent	160 (30.1)
What do you think about ultrasound examination? *	
Painful	18 (3.4)
Not comfortable	120 (22.6)
Comfortable	405 (76.3)
Where did you get the majority of the information about your examination? *	
Family and Friends	149 (28.1)
Internet and Social media	165 (31.1)
Referring Doctor	311 (58.6)
Radiology Clinical Staff	198 (37.3)
Other	86 (16.2)

* Multiple responses.

**Table 4:** Patient characteristics related to their ultrasound knowledge level by bivariate analysis.

Characteristics	Knowledge Level of Ultrasound		χ^2^ Value	*p*-Value	Unadjusted Odds Ratio (95% CI)
	Adequate (*n* = 397)	Inadequate (*n* = 134)			
Age groups(in years)					
Less than 20	4 (36.4)	7 (63.6)	13.05	0.005	1.0 (ref)
20–29	101 (76.5)	31 (23.5)			14.06 (2.89, 68.48)
30–39	172 (79.3)	45 (20.7)			16.28 (3.40, 77.91)
40+	120 (70.2)	51 (29.8)			9.70 (2.04, 46.66)
Institution					
Private	194 (75.5)	63 (24.5)	0.138	0.711	1.0 (ref.)
Public	203 (74.1)	71 (25.9)			1.25 (0.85, 1.83)
Education					
None	10 (76.9)	3 (23.1)	12.90	0.012	1.0 (ref.)
Primary, Intermediate, and Secondary	75 (62.5)	45 (37.5)			9.17 (1.94, 43.24)
Diploma	26 (81.3)	6 (18.8)			23.83 (4.15, 136.97)
BSc	242 (77.6)	70 (22.4)			19.01 (4.12, 87.81)
MSc and PhD	44 (81.5)	10 (18.5)			24.20 (4.62, 126.73)
Employment					
Student	19 (79.2)	5 (20.8)	6.98	0.222	2.70 (0.64, 9.81)
Unemployed	40 (78.4)	11 (21.6)			2.92 (0.96, 8.86)
Housewife	138 (72.3)	53 (27.7)			2.01 (0.78, 5.21)
Freelance	13 (76.5)	4 (23.5)			2.92 (0.69, 12.32)
Employed	177 (77.3)	52 (22.7)			3.06 (1.18, 7.94)
Retired	10 (52.6)	9 (47.4)			1.0 (ref.)
Trimester Stage					
Don’t know	140 (72.9)	52 (27.1)	3.04	0.385	1.0 (ref.)
First	43 (69.4)	19 (30.6)			0.91 (0.49, 1.68)
Second	91 (74.6)	31 (25.4)			1.22 (0.73, 2.02)
Third	123 (79.4)	32 (20.6)			1.66 (1.01, 2.73)
Ultrasound before this pregnancy					
Yes	331 (80.7)	79 (19.3)	33.96	<0.0001	3.25 (2.11, 4.98)
No	66 (54.5)	55 (45.5)			1.0 (ref.)
First pregnancy					
Yes	82 (65.1)	44 (34.9)	8.21	0.004	1.0 (ref.)
No	315 (77.8)	90 (22.2)			1.76 (1.15, 2.71)

**Table 5:** Independent variables associated with ultrasound knowledge level (adequate) by multivariate binary logistic regression.

Variables	B	Standard Error	Wald Statistics	Adjusted Odds Ratios (95% CI)	*p*-Value
Age group (in years)					
Less than 20	--	--	--	1.0 (ref.)	--
20–29	1.848	0.856	4.666	6.35 (1.19, 33.95)	0.031
30–39	1.637	0.862	3.607	5.14 (0.95, 27.82)	0.058
40+	1.162	0.879	1.747	3.20 (0.57, 17.91)	0.186
Education					
None	--	--	--	1.0 (ref.)	--
Primary, Intermediate, and Secondary	1.987	0.823	5.834	7.30 (1.45, 36.60)	0.016
Diploma	3.116	0.929	11.238	22.55 (3.65, 139.37)	0.001
BSc	2.641	0.815	10.505	14.03 (2.84, 69.26)	0.001
MSc and PhD	2.941	0.889	10.955	18.94 (3.32, 108.11)	0.001
Ultrasound before this pregnancy					
Yes	0.950	0.245	15.056	2.58 (1.60, 4.18)	<0.0001
No	--	--	--	1.0 (ref.)	--
First pregnancy					
Yes	--	--	--	1.0 ( ref.)	--
No	0.583	0.293	3.953	1.79 (1.01, 3.18)	0.047
Model χ^2^ = 69.410 (*p* < 0.001) Nagelkerke Pseudo R^2^ = 0.18 Goodness of fit using Hosmer and Lemeshow = 7.495 (*p* = 0.484)					

**Table 6:** Association between participants’ self-evaluation and knowledge level of ultrasound.

Self-Evaluation	Knowledge Level: No. (%) *		Total (%) **	χ^2^ Value	*p*-Value
	Adequate	Inadequate			
Not enough	12 (23.1)	40 (76.9)	52 (9.8)	106.034	<0.0001
Fair	78 (62.4)	47 (37.6)	125 (23.5)		
Enough	36 (73.5)	13 (26.5)	49 (9.2)		
Good	116 (80.0)	29 (20.0)	145 (27.3)		
Excellent	147 (91.9)	13 (8.1)	160 (30.1)		

* Row %; ** Column %.

## Data Availability

A full data set is available upon request to the corresponding author.
